# Syntheses, crystal structures and Hirshfeld surface analyses of bis­(2-mercaptobenzimidazole)­bromo- and iodo­copper(I) complexes

**DOI:** 10.1107/S2056989022004224

**Published:** 2022-04-26

**Authors:** Pipat Chooto, Saowanit Saithong, Weena Aemaeg, Siriwan Vataporn, Chaveng Pakawatchai, Chalermpol Innuphat, Supunnee Duangthong, Walailak Puetpaiboon

**Affiliations:** a Department of Chemistry, Faculty of Science, Prince of Songkla University, Hatyai, Songkhla 90112, Thailand; b Division of Physical Science and Center of Excellence for Innovation in Chemistry, Faculty of Science, Prince of Songkla, University, Hat Yai, Songkhla 90112, Thailand; c Division of Physical Science, Faculty of Science, Prince of Songkla University, Hatyai, Songkhla 90112, Thailand

**Keywords:** 2-mercaptobenzimidazole, copper, crystal structure

## Abstract

The title complexes, bromidobis(2,3-dihydro-1*H*-1,3-benzodiazole-2-thi­one)copper(I), [CuBr(C_7_H_6_N_2_S)_2_] (**1**), and bis(2,3-dihydro-1*H*-1,3-benzodiazole-2-thione)iodidocopper(I) acetone monosolvate, [CuI(C_7_H_6_N_2_S)_2_]·CH_3_COCH_3_ (**2**), were prepared by the reaction of copper(I) bromide/iodide with 2-mercaptobenzimidazole. Both complexes have mononuclear structures with the copper atom coordinated by two 2-mercaptobenzimidazole mol­ecules *via* their S atoms and one halide atom in an approximate trigonal–planar arrangement. In their extended structures, N—H⋯S hydrogen bonds and π–π contacts are found in both complexes; as a result of the acetone solvent mol­ecule in (**2**), N—H⋯O contacts are also observed. Hirshfeld surface analyses were carried out to aid in the visualization of these inter­actions, which showed that H⋯H contacts contribute 34.6% for (**1**) and 34.1% for (**2**) to the overall surface, followed by contributions from H⋯S/S⋯H, H⋯C/C⋯H and C⋯C contacts, respectively. As expected, H⋯O/O⋯H contacts are observed only in (**2**). The IR and ^1^H and ^13^C NMR spectra of (**1**) and (**2**) are described.

## Abstract 2

The title complexes feature distorted trigonal–planar CuS_2_ *X* (*X* = Br, I) coordination geometries for the metal ions. The presence of the acetone solvent mol­ecule in the iodide complex plays an important role in the packing.

## 1. Chemical context

2-Mercaptobenzimidazole (C_7_H_6_N_2_S; bimztH_2_) has many uses including as an anti­oxidant to prevent rubber deterioration (Moldovan & Alexandrescu, 2002 ▸), an absorbant of mercury from industrial waste water in the form of 2-mercaptobenzimidazole-clay (Manohar *et al.*, 2002 ▸), as a modifier of electrode surfaces to increase the efficiency of electrochemical analysis (Berchmans *et al.*, 2000 ▸), as an inter­mediate in the production of the anti-inflammatory drug lanzoprazole (Wongwattana, 2004 ▸) and as a Cu corrosion inhibitor (Finšgar, 2013 ▸).

The preparation of bimztH_2_ involves the reaction between *o*-phenyl­enedi­amine and potassium ethyl xanthate in an ethanol–water mixture followed by reaction with acetic acid and water at 333–343 K (Vanallan & Deacon, 1971 ▸). The structure of bimztH_2_ exhibits tautomerism between its thione and thiol forms (Rout *et al.*, 1984 ▸) as shown in the scheme below. 
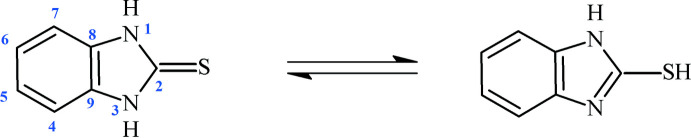


We now describe the syntheses and crystal structures of bimztH_2_ complexes with copper(I) halides, Cu*X* (*X* = Br, I). It may be noted that the S atom of the ligand is a soft base and therefore favoured to form a coordinate bond with a soft acid such as copper(I). Hirshfeld surface analyses were performed to gain further insight into the inter­molecular inter­actions in these structures. 
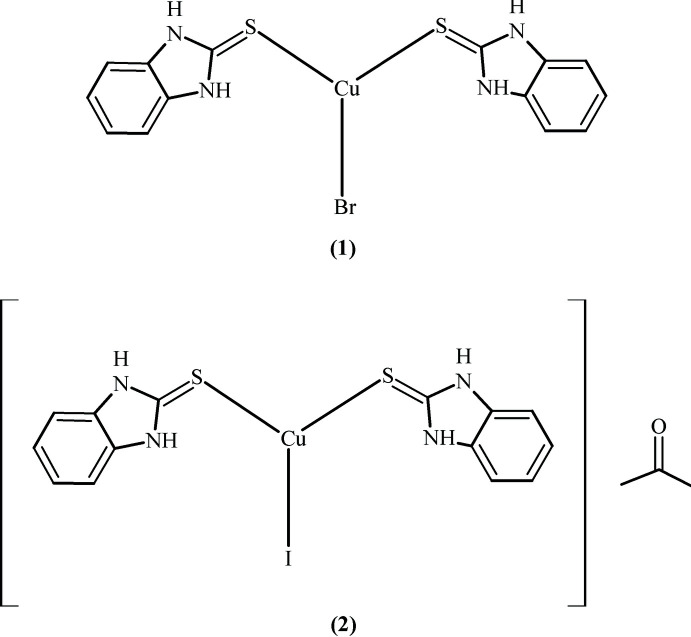


## 2. Structural commentary

**Figure 1:**
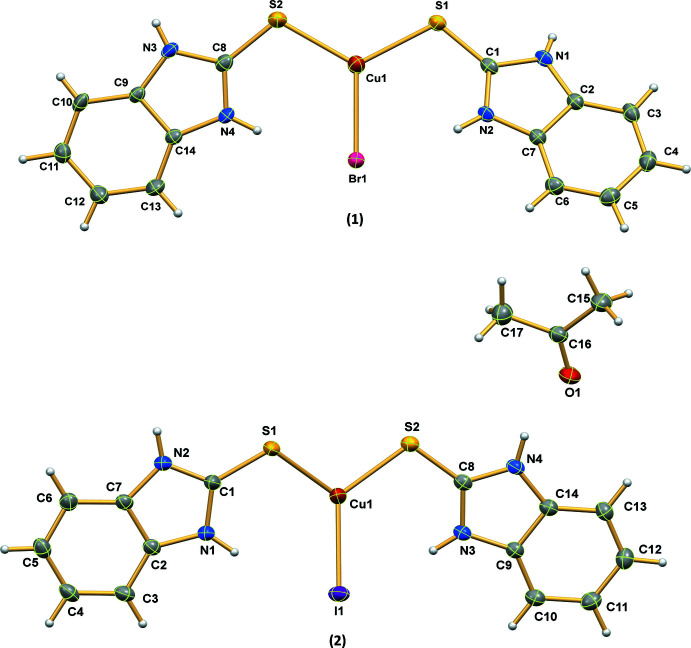
The mol­ecular structures of (**1**) and (**2**) showing **xx% [please supply]** displacement ellipsoids.

The mononuclear structures of [Cu(bimztH_2_)_2_Br] (**1**) and [Cu(bimztH_2_)_2_I]·CH_3_COCH_3_ (**2**) are depicted in Fig. 1 ▸. Both complexes crystallize in the monoclinic system, space group *P*2_1_/*c*. The copper ions adopt distorted trigonal–planar coord­ination geometries with one Cu—*X* bond (*X* = Br, I) and two Cu—S bonds, the lengths of which lie between 2.2189 (15) and 2.5479 (7) Å, being close to those found in complexes with a trigonal–planar geometry such as [Cu_2_(mimtH)_5_]^2+^ (Atkinson *et al.*, 1985 ▸) and [Cu(SC_6_H_5_)_3_]^2−^ (Coucouvanis *et al.*, 1980 ▸). When comparing (**1**) and (**2**), the bond angles are distorted from the ideal values of 120° with greater distortion in (**2**) resulting from the presence of the acetone solvent mol­ecule and an N4—H4*A*⋯O1 hydrogen bond. The acetone mol­ecules in (**2**) result in weaker C=S bonds as supported by IR and ^13^C NMR data (*vide infra*). Both complexes feature a pair of intra­molecular N—H⋯*X* hydrogen bonds as listed in Tables 1 ▸ and 2 ▸ for (**1**) and (**2**), respectively.

## 3. Supra­molecular features

**Figure 2:**
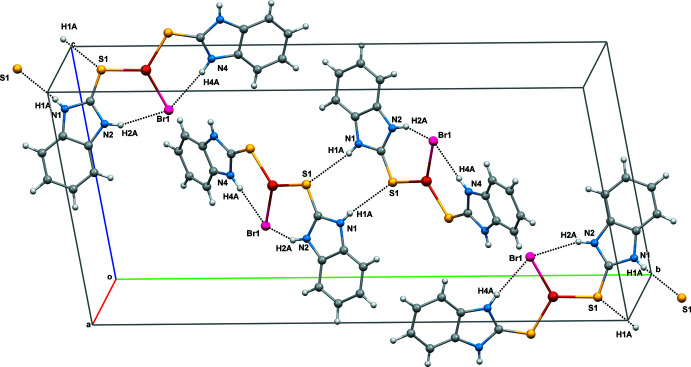
The intra- and inter mol­ecular hydrogen-bonding inter­actions of (**1**).

**Figure 4:**
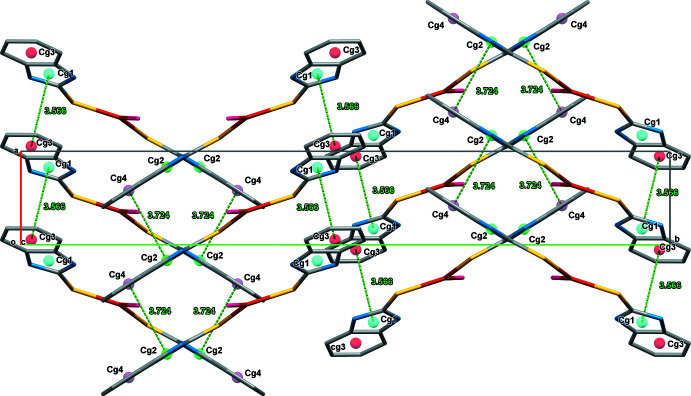
The inter­molecular π–π inter­actions in the crystal packing of (**1**) plotted down the *c* axis.

**Figure 5:**
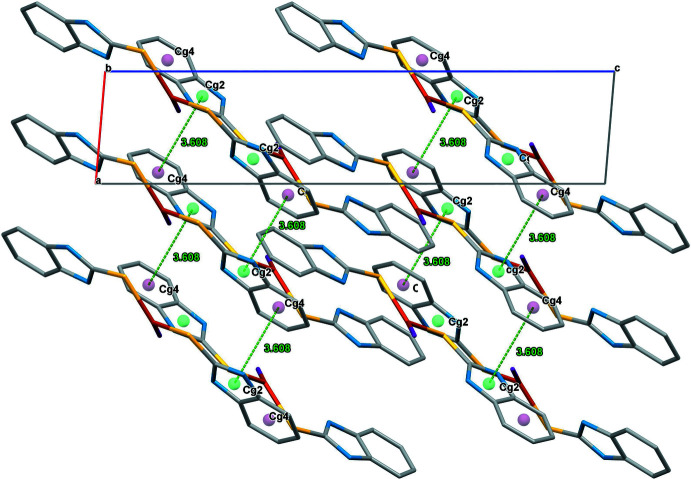
The inter­molecular π–π inter­actions in the crystal packing of (**2**) plotted down the *b* axis.

The supra­molecular assemblies in (**1**) and (**2**) (Tables 1 ▸ and 2 ▸) feature pairwise N—H⋯S hydrogen bonds, generating a graph-set 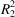 (8) pattern with N1⋯S1^i^ = 3.384 (4) Å for (**1**) and N2⋯S1^i^ = 3.331 (4) Å for (**2**) [symmetry code: (i) −*x* + 1, −*y* + 1, −*z* + 1 for (**1**) and −*x* − 1, −*y* + 1, −*z* + 1 for (**2**)]. The acetone solvent mol­ecule in (**2**) leads to the formation of an N4—H4*A*⋯O1 hydrogen bond with N4⋯O1 = 2.840 (5) Å. The intra- and inter­molecular hydrogen-bond contacts of (**1**) and (**2**) are shown in Figs. 2 ▸ and 3 ▸, respectively. In addition, aromatic π–π stacking contacts are observed between adjacent imidazole rings (*Cg1*: N1/C1/N2/C7/C2 and *Cg2*: N3/C8/N4/C14/C9) and phenyl rings (*Cg3*: C2–C7 and *Cg4*: C9–C14) of neighbouring complex mol­ecules. The π–π inter­actions in the packing of (**1**) occur between *Cg1*–*Cg3* (set 1) and *Cg2*–*Cg4* (set 2) as inter-digitated [100] stacks with a minimum centroid–centroid separation of 3.566 (3) Å (Fig. 4 ▸), while in the packing of (**2**) (Fig. 5 ▸), corresponding *Cg2*–*Cg4* inter­actions occur, which also leads to [100] stacks [minimum centroid–centroid separation = 3.608 (3) Å].

## 4. Hirshfeld surface analysis

**Figure 6:**
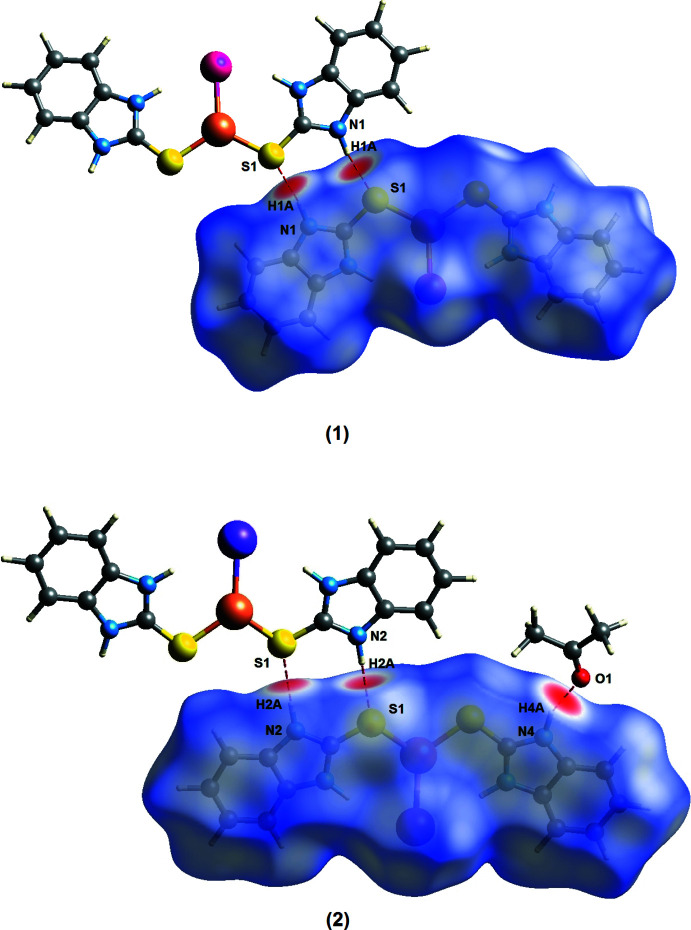
Hirshfeld surfaces plotted over *d*_norm_ showing the areas of inter­molecular hydrogen-bonding contacts of (**1**) and (**2**).

**Figure 7:**
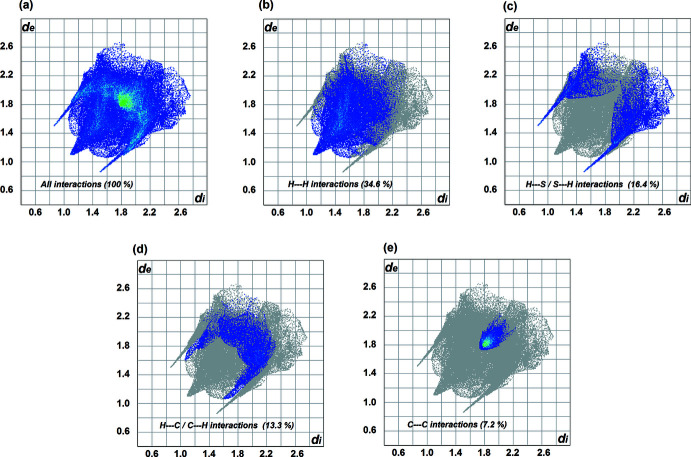
The fingerprint plots for (**1**).

**Figure 8:**
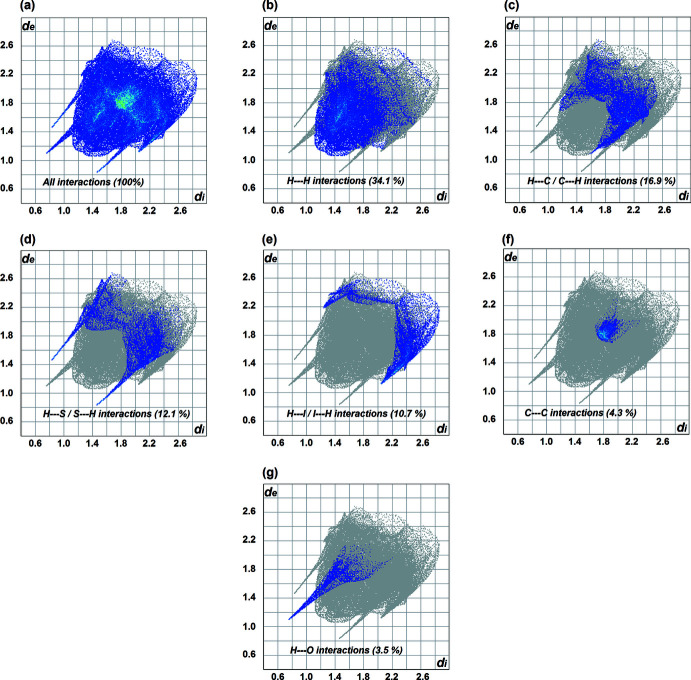
The fingerprint plots for (**2**).

The Hirshfeld surface (HS) analyses (HS mapped over *d*_norm_ are shown in Fig. 6 ▸) and *d_e_* and *d_i_* fingerprint plots (Figs. 7 ▸ and 8 ▸) were generated using *Crystal Explorer 17.5* (Turner *et al.*, 2017 ▸). The red spots indicate the donors and acceptors of the hydrogen bonds, appearing close to H1*A* and S1 of the N1—H1*A*⋯S1 bond for (**1**) and close to H2*A*⋯S1 of the N2*A-*–H2*A*⋯S1 bond for (**2**). In addition, a red spot is found between H4*A* and O1 of the acetone solvent mol­ecule for (**2**). The fingerprint plots for (**1**) show that the principal inter­molecular contacts are H⋯H at 34.6% (Fig. 7 ▸ *b*), H⋯S /S⋯H at 16.4% (Fig. 7 ▸ *c*), H⋯C/C⋯H at 13.3% (Fig. 7 ▸ *d*) and C⋯C contacts at 7.2% (Fig. 7 ▸ *e*). For complex (**2**), the principal contacts are H⋯H at 34.1% (Fig. 8 ▸ *b*), H⋯C/C⋯H at 16.9% (Fig. 8 ▸ *c*), H⋯S / S⋯H at 12.1% (Fig. 8 ▸ *d*) and C⋯C contacts at 4.3% (Fig. 8 ▸ *e*) followed by H⋯O contacts at 3.5% (Fig. 8 ▸ *f*). As can be seen, H⋯H contacts predominate in both complexes, followed by H⋯S/S⋯H contacts for (**1**) and H⋯C/C⋯H contacts for (**2**). However, the C⋯C contacts differ significantly (by 3.7%) indicating that the π–π inter­molecular inter­actions in (**1**) are stronger than in (**2**).

## 5. Database survey

2-Mercaptobenzimidazole has been found to form a complex with Pt, the bond formation being *via* the sulfur atom only with a square-planar geometry [Cambridge Structural Database (Groom *et al.*, 2016 ▸) refcode GURMOV; Jolley *et al.*, 2001 ▸]. In the case of the Co^II^ complex, two sulfur atoms are bonded with the metal atom in a tetra­hedral coordination geometry (refcode ZOKYAZ; Ravikumar *et al.*, 1995 ▸). Cu^I^ complexes with 2-mercaptobenzimidazole derivatives have been investigated as a model of copper proteins (refcodes QORGUZ, QORHAG and QORHEK; Balamurugan *et al.*, 2001 ▸). A series of polynuclear clusters containing Ni^II^ and Co^II^ (refcodes FOPVEN, FOPVIR and FOPXOZ; Han *et al.*, 2015 ▸) of this ligand have been synthesized and the magnetic susceptibility of an Ni^II^ complex (FOPVEN) has been reported. The photophysical properties of the rigid structure of a hexa­nuclear Cu^I^ complex of 2-mercaptobenzimidazole constructed by S bridges has been studied (refcode COPNUT; Singh *et al.*, 2017 ▸).

## 6. Synthesis, crystallization and chracterization

**[Cu(bimztH_2_)_2_Br] (1)**

A mass of 0.19 g (1.2 mmol) of bimztH_2_ was placed in 30 ml of acetone at 318 K and stirred until completely dissolved to form a colourless solution. CuBr (0.09 g; 0.6 mmol) was added followed by further stirring for about 15 min to obtain a yellow solution, which was refluxed for 120 min at 353 K to become turbid with a light-yellow colour and then filtered. The colourless filtrate was left at room temperature for 3 days to form transparent needles and then filtered by vacuum suction to obtain 0.16 g of (**1**) (58% yield, m.p. 518–523 K). Elemental analysis (%): found (calculated); C = 38.32 (37.86), H = 2.78 (2.73), N = 12.17 (12.62), S = 14.71 (14.45).

**[Cu(bimztH_2_)_2_I]·CH_3_COCH_3_ (2)**

The same procedure for (**1**) was followed except that 0.060 g of CuI (1.6 mmol) replaced the CuBr and 0.21 g of colourless needles of (**2**) were recovered (73%, yield, m.p. 518–523 K). Elemental analysis (%); found (calculated): C = 38.32 (37.86), H = 2.78 (2.73), N = 12.17 (12.62), S = 14.71 (14.45).

**FT–IR spectra**

Suzuki (1962 ▸) proposed that features in thio­amide IR spectra could be assigned to band I at 1395–1570 cm^−1^ arising from the N—H deformation and C—N stretching; band II at 1270–1420 cm^−1^ from C—N stretching, N—H deformation and C—H bending, band III at 940–1140 cm^−1^ from C—N and C=S stretching and band IV at 680–860 cm^−1^ due to C=S stretching (compare Jolley *et al.*, 2001 ▸). Additionally, Raper *et al.* (1988 ▸) studied absorption bands of thio­amide in the complex prepared from bimzH_2_ and copper(II) perchlorate and found them at 1470 cm^−1^ (band I), 1360 cm^−1^ (band II), 1180 cm^−1^ (band III) and 740 cm^−1^ (band IV) compared with those of the free ligand at 1468 cm^−1^, 1357 cm^−1^, 1181 cm^−1^ and 744 + 713 cm^−1^, respectively. The broad absorption band at 3155 cm^−1^ is due to N—H stretching, which moves to a higher wavenumber and splits into two upon complexation.

For all our complexes, the FT–IR spectrum indicates the shift of bands I and II to a higher wavenumber, similar to the behaviour of N—H stretching due to the coordination through the sulfur atom and resulting charge transfer from N to S, which makes the N—H and C—N bonds stronger (Aslanidis *et al.*, 2002 ▸). Band III of thio­amide for all complexes shifts to a lower wavenumber but this is hard to qu­antify because this area also covers C—N stretching. Band IV for C=S stretching changes significantly from 744 and 713 cm^−1^ in the free ligand to 734 cm^−1^ in the complex, reflecting copper–sulfur coordination. A change also occurs for the C—S bending mode at 602 cm^−1^ of C—S bending to lower wavenumber, corresponding with previous work (Raper *et al.*, 1988 ▸). In the case of [Cu(bimztH_2_)_2_I]·CH_3_COCH_3_, the absorption bands at 1688 and 1384 cm^−1^ were found (figure not shown). After heating at 383 K for 10 minutes, these bands disappeared. Therefore these are due to C=O stretching and C—H bending, respectively, indicating the presence of acetone in the compound. IR data are summarized in Table 3 ▸.

**^1^H NMR and ^13^C NMR spectra**

^1^H NMR data for the ligand and (**1**) and (**2**) are listed in Table 4 ▸. The chemical shift at 13.28 ppm (*br*, *s*) belongs to two groups of N—H protons. The ratio of integration reveals that the two protons have the same environment. The ratio of N—H and aromatic protons is 1:2 without the signal of the S—H proton, indicating that both ligand and complex contain thione in DMSO-*d*_6_ (Isab *et al.*, 2003 ▸). Furthermore, the ligand exhibits chemical shifts around 7.49 ppm due to four methane protons on an aromatic benzene ring at positions H_4_, H_7_, H_5_, and H_6_, which change upon complex formation. The ^13^C NMR spectra of the ligand and complexes (Table 5 ▸) reveal seven carbon signals, including that of the thio­carbonyl group at 168.34 ppm, four carbon atoms in the aromatic ring at 109.75 and 122.59 ppm for C_4,7_ and C_5,6_, respectively, and two quarternary carbon atoms at 132.48 ppm. In the complex, C_2_ and C_8,9_ have upfield chemical shifts due to more electron shielding. The coordination *via* sulfur causes C=S to be weaker as well as the electron density to change from nitro­gen to C_2_, whereas C_4,7_ and C_5,6_ have downfield chemical shifts due to the electron transfer to C_8,9_, corresponding with the work of Isab *et al.* (2003 ▸). For (**2**), the carbonyl signal at 206.64 ppm and methane carbon at 30.86 ppm indicate the presence of acetone in the compound.

## 7. Refinement

Crystal data, data collection and structure refinement details are summarized in Table 6 ▸. All H atoms of (**1**) were clearly resolved in difference-density maps and all H-atom parameters were freely refined. For (**2**), the carbon-bound H atoms were placed in calculated locations with C—H = 0.93–0.96 Å and refined as riding atoms with *U*_iso_(H) = 1.2*U*_eq_(C) or 1.5*U*_eq_(methyl C). The H-atom positions of the amide groups of (**2**) were found in difference maps and refined with N—H distances restained to 0.85 (2) and 0.86 (2) Å.

## Supplementary Material

Crystal structure: contains datablock(s) 1, 2, global. DOI: 10.1107/S2056989022004224/hb7984sup1.cif

Structure factors: contains datablock(s) 1. DOI: 10.1107/S2056989022004224/hb79841sup2.hkl

Structure factors: contains datablock(s) 2. DOI: 10.1107/S2056989022004224/hb79842sup3.hkl

CCDC references: 2167595, 2167594

Additional supporting information:  crystallographic information; 3D view; checkCIF report

## Figures and Tables

**Figure 3:**
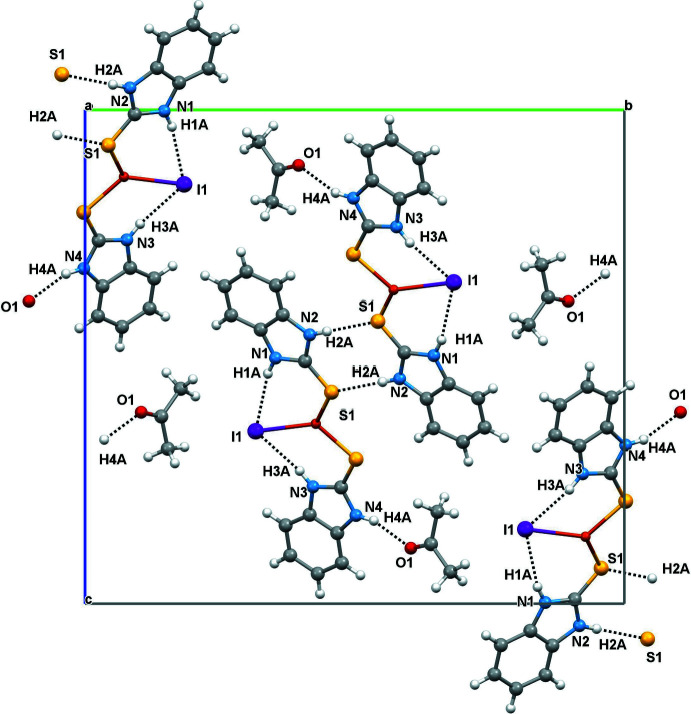
The intra- and inter mol­ecular hydrogen-bonding inter­actions of (**2**).

**Table 1:** Hydrogen-bond geometry (Å, °) for (1)

*D*—H⋯*A*	*D*—H	H⋯*A*	*D*⋯*A*	*D*—H⋯*A*
N1—H1*A*⋯S1^i^	0.70 (4)	2.69 (4)	3.384 (4)	170 (5)
N2—H2*A*⋯Br1	0.82 (4)	2.65 (4)	3.361 (4)	146 (4)
N4—H4*A*⋯Br1	0.84 (4)	2.54 (4)	3.364 (4)	166 (4)

Symmetry code: (i) 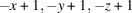 .

**Table 2:** Hydrogen-bond geometry (Å, °) for (2)

*D*—H⋯*A*	*D*—H	H⋯*A*	*D*⋯*A*	*D*—H⋯*A*
N1—H1*A*⋯I1	0.86 (2)	2.81 (2)	3.649 (4)	167 (6)
N2—H2*A*⋯S1^i^	0.86 (2)	2.47 (2)	3.331 (4)	176 (6)
N3—H3*A*⋯I1	0.85 (2)	2.91 (4)	3.666 (3)	148 (5)
N4—H4*A*⋯O1	0.85 (2)	2.02 (3)	2.840 (5)	161 (6)

Symmetry code: (i) 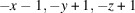 .

**Table 3:** IR peak assignments (cm^−1^) for the bimztH2 ligand and (**1**) and (**2**)

Compound	ν(N—H)	Thio­amide band I	Thio­amide band II	Thio­amide band III	Thio­amide band IV	δ (C=S)
bimztH2	3155	1468	1357	1181	744, 713	602
(**1**)	3201, 3383	1470	1360	1180	734	598
(**2**)	3202, 3385	1470	1361	1175	734	598

**Table 4:** ^1^H NMR chemical shifts (p.p.m.) of the bimztH_2_ ligand, (**1**) and (**2**)

Compound	H4, H7	H5, H6	N—H
bimztH_2_	7.49 (4*H*, *m*)	7.49 (4*H*, *m*)	13.28 (*br*, *s*)
(**1**)	7.26 (2*H*, *dd*, *J* = 6.3 Hz)	7.19 (2*H*, *dd*, *J* = 5.5 Hz)	12.87 (*br*, *s*)
(**2**)	7.58 (2*H*, *s*)	7.58 (2*H*, *s*)	13.57 (*br*, *s*)

**Table 5:** ^13^C NMR chemical shifts (p.p.m.) of the bimztH2 ligand and (**1**) and (**2**)

Compound	C2 (C=S)	C_4,7_ (CH)	C_5,6_ (CH)	C_8,9_ (C)
bimztH2	168.34	109.75	122.59	132.48
(**1**)	165.11	110.35	123.06	132.06
(**2**)	164.10	110.65	123.29	131.96

**Table 6:** Experimental details

	(**1**)	(**2**)
Crystal data		
Chemical formula	[CuBr(C_7_H_5_N_2_S)_2_]	[CuI(C_7_H_6_N_2_S)_2_]·C_3_H_6_O
*M*_r_	443.85	548.91
Crystal system, space group	Monoclinic, *P*2_1_/*c*	Monoclinic, *P*2_1_/*c*
Temperature (K)	293	293
*a*, *b*, *c* (Å)	4.1549 (4), 28.708 (3), 13.2735 (13)	4.5154 (3), 22.2157 (15), 20.4062 (14)
β (°)	95.564 (2)	94.818 (1)
*V* (Å^3^)	1575.8 (3)	2039.8 (2)
*Z*	4	4
Radiation type	Mo *K*α	Mo *K*α
μ (mm^−1^)	4.19	2.80
Crystal size (mm)	0.46 × 0.05 × 0.04	0.38 × 0.14 × 0.08

Data collection		
Diffractometer	Bruker CCD area detector	Bruker CCD area detector
Absorption correction	Multi-scan (*SADABS*; Bruker, 2003 ▸)	Multi-scan (*SADABS*; Bruker, 2003 ▸)
*T*_min_, *T*_max_	0.713, 1.000	0.749, 1.000
No. of measured, independent and observed [*I* > 2σ(*I*)] reflections	11218, 2750, 2200	14555, 3597, 3200
*R*_int_	0.039	0.021
(sin θ/λ)_max_ (Å^−1^)	0.595	0.595

Refinement		
*R*[*F* ^2^ > 2σ(*F* ^2^)], *wR*(*F* ^2^), *S*	0.041, 0.093, 1.08	0.039, 0.089, 1.08
No. of reflections	2750	3597
No. of parameters	247	247
No. of restraints	0	4
H-atom treatment	All H-atom parameters refined	H atoms treated by a mixture of independent and constrained refinement
Δρ_max_, Δρ_min_ (e Å^−3^)	0.66, −0.39	1.14, −0.90

Computer programs: *SMART* and *SAINT* (Bruker, 2003 ▸), *SHELXT* (Sheldrick, 2015*a* ▸), *SHELXL2018/3* (Sheldrick, 2015*b* ▸), *Mercury* (Macrae *et al.*, 2020 ▸), *WinGX* publication routines (Farrugia, 2012 ▸) and *publCIF* (Westrip, 2010 ▸).

## supplementary crystallographic information

### Bromidobis(2,3-dihydro-1H-1,3-benzodiazole-2-thione)copper(I) (1) Crystal data

**Table:**

[CuBr(C_7_H_5_N_2_S)_2_]	*F*(000) = 880
*M**_r_* = 443.85	*D*_x_ = 1.871 Mg m^−^^3^
Monoclinic, *P*2_1_/*c*	Mo *K*α radiation, λ = 0.71073 Å
*a* = 4.1549 (4) Å	Cell parameters from 2031 reflections
*b* = 28.708 (3) Å	θ = 2.6–21.8°
*c* = 13.2735 (13) Å	µ = 4.19 mm^−^^1^
β = 95.564 (2)°	*T* = 293 K
*V* = 1575.8 (3) Å^3^	Needle, colourless
*Z* = 4	0.46 × 0.05 × 0.04 mm

### Bromidobis(2,3-dihydro-1H-1,3-benzodiazole-2-thione)copper(I) (1) Data collection

**Table:**

Bruker CCD area detector diffractometer	2750 independent reflections
Radiation source: fine-focus sealed tube	2200 reflections with *I* > 2σ(*I*)
Graphite monochromator	*R*_int_ = 0.039
Frames, each covering 0.3 ° in ω scans	θ_max_ = 25.0°, θ_min_ = 1.7°
Absorption correction: multi-scan (SADABS; Bruker, 2003)	*h* = −4→4
*T*_min_ = 0.713, *T*_max_ = 1.000	*k* = −34→33
11218 measured reflections	*l* = −15→15

### Bromidobis(2,3-dihydro-1H-1,3-benzodiazole-2-thione)copper(I) (1) Refinement

**Table:**

Refinement on *F*^2^	Primary atom site location: structure-invariant direct methods
Least-squares matrix: full	Secondary atom site location: difference Fourier map
*R*[*F*^2^ > 2σ(*F*^2^)] = 0.041	Hydrogen site location: difference Fourier map
*wR*(*F*^2^) = 0.093	All H-atom parameters refined
*S* = 1.08	*w* = 1/[σ^2^(*F*_o_^2^) + (0.0446*P*)^2^ + 0.5475*P*] where *P* = (*F*_o_^2^ + 2*F*_c_^2^)/3
2750 reflections	(Δ/σ)_max_ < 0.001
247 parameters	Δρ_max_ = 0.66 e Å^−^^3^
0 restraints	Δρ_min_ = −0.39 e Å^−^^3^

### Bromidobis(2,3-dihydro-1H-1,3-benzodiazole-2-thione)copper(I) (1) Special details

**Table:**

Geometry. All esds (except the esd in the dihedral angle between two l.s. planes) are estimated using the full covariance matrix. The cell esds are taken into account individually in the estimation of esds in distances, angles and torsion angles; correlations between esds in cell parameters are only used when they are defined by crystal symmetry. An approximate (isotropic) treatment of cell esds is used for estimating esds involving l.s. planes.

### Bromidobis(2,3-dihydro-1H-1,3-benzodiazole-2-thione)copper(I) (1) Fractional atomic coordinates and isotropic or equivalent isotropic displacement parameters (Å^2^)

**Table:**

	*x*	*y*	*z*	*U*_iso_*/*U*_eq_	
Br1	0.35082 (11)	0.31949 (2)	0.29213 (3)	0.04740 (17)	
Cu1	0.35888 (16)	0.34728 (2)	0.46565 (5)	0.0570 (2)	
S1	0.5237 (3)	0.42049 (4)	0.50112 (9)	0.0489 (3)	
N1	0.7906 (10)	0.48624 (13)	0.3871 (3)	0.0439 (10)	
C1	0.7004 (10)	0.44151 (14)	0.4012 (3)	0.0399 (10)	
S2	0.1419 (4)	0.30563 (5)	0.58260 (10)	0.0688 (4)	
N2	0.7887 (9)	0.41786 (13)	0.3211 (3)	0.0414 (9)	
C2	0.9318 (10)	0.49139 (15)	0.2975 (3)	0.0407 (10)	
N3	−0.1802 (11)	0.22338 (14)	0.5747 (3)	0.0527 (11)	
C3	1.0529 (12)	0.52902 (17)	0.2500 (4)	0.0545 (13)	
N4	−0.0687 (9)	0.24669 (13)	0.4289 (3)	0.0444 (9)	
C4	1.1761 (13)	0.52084 (19)	0.1592 (4)	0.0595 (14)	
C5	1.1740 (12)	0.47661 (19)	0.1173 (4)	0.0572 (13)	
C6	1.0519 (12)	0.43907 (19)	0.1639 (4)	0.0528 (12)	
C7	0.9299 (10)	0.44709 (15)	0.2558 (3)	0.0395 (10)	
C8	−0.0348 (11)	0.25789 (15)	0.5269 (3)	0.0463 (11)	
C9	−0.3156 (10)	0.19077 (14)	0.5067 (3)	0.0393 (10)	
C10	−0.4919 (13)	0.15060 (17)	0.5169 (4)	0.0530 (13)	
C11	−0.5958 (13)	0.12685 (18)	0.4308 (4)	0.0560 (13)	
C12	−0.5244 (14)	0.14225 (19)	0.3371 (4)	0.0623 (14)	
C13	−0.3471 (14)	0.18181 (19)	0.3265 (4)	0.0610 (14)	
C14	−0.2427 (10)	0.20588 (15)	0.4124 (3)	0.0411 (10)	
H1A	0.734 (10)	0.5041 (15)	0.417 (3)	0.032 (13)*	
H2A	0.764 (10)	0.3900 (16)	0.310 (3)	0.049 (14)*	
H3	1.054 (10)	0.5568 (15)	0.282 (3)	0.045 (12)*	
H3A	−0.203 (10)	0.2208 (15)	0.631 (3)	0.038 (14)*	
H4	1.259 (12)	0.5470 (17)	0.126 (4)	0.069 (16)*	
H4A	0.008 (9)	0.2643 (14)	0.387 (3)	0.033 (11)*	
H5	1.263 (12)	0.4737 (16)	0.057 (4)	0.062 (15)*	
H6	1.045 (12)	0.4107 (18)	0.139 (4)	0.073 (17)*	
H10	−0.542 (11)	0.1438 (16)	0.573 (4)	0.051 (14)*	
H11	−0.727 (13)	0.0991 (19)	0.436 (4)	0.082 (18)*	
H12	−0.591 (11)	0.1243 (17)	0.284 (4)	0.065 (16)*	
H13	−0.285 (11)	0.1901 (16)	0.269 (4)	0.052 (14)*	

### Bromidobis(2,3-dihydro-1H-1,3-benzodiazole-2-thione)copper(I) (1) Atomic displacement parameters (Å^2^)

**Table:**

	*U*^11^	*U*^22^	*U*^33^	*U*^12^	*U*^13^	*U*^23^
Br1	0.0583 (3)	0.0418 (3)	0.0432 (3)	−0.0072 (2)	0.0106 (2)	−0.0012 (2)
Cu1	0.0745 (5)	0.0403 (3)	0.0586 (4)	−0.0038 (3)	0.0192 (3)	−0.0036 (3)
S1	0.0628 (8)	0.0383 (6)	0.0469 (7)	0.0016 (5)	0.0115 (6)	−0.0075 (5)
N1	0.050 (2)	0.030 (2)	0.051 (3)	0.0076 (18)	0.0024 (19)	−0.0082 (19)
C1	0.035 (2)	0.033 (2)	0.049 (3)	0.0086 (19)	−0.0047 (19)	−0.006 (2)
S2	0.1016 (11)	0.0582 (8)	0.0498 (8)	−0.0207 (8)	0.0242 (7)	−0.0117 (6)
N2	0.050 (2)	0.028 (2)	0.047 (2)	0.0024 (17)	0.0089 (17)	−0.0078 (17)
C2	0.039 (2)	0.037 (2)	0.045 (3)	0.0032 (19)	−0.002 (2)	−0.001 (2)
N3	0.077 (3)	0.046 (2)	0.037 (3)	−0.001 (2)	0.022 (2)	0.006 (2)
C3	0.062 (3)	0.035 (3)	0.064 (3)	−0.002 (2)	−0.005 (3)	0.001 (2)
N4	0.055 (2)	0.044 (2)	0.035 (2)	−0.0042 (19)	0.0065 (18)	0.0093 (18)
C4	0.058 (3)	0.058 (3)	0.063 (4)	−0.009 (3)	0.003 (3)	0.015 (3)
C5	0.049 (3)	0.069 (4)	0.055 (3)	0.002 (3)	0.011 (3)	0.001 (3)
C6	0.053 (3)	0.048 (3)	0.058 (3)	0.003 (2)	0.008 (2)	−0.005 (3)
C7	0.035 (2)	0.038 (2)	0.045 (3)	0.0021 (18)	0.0008 (19)	−0.003 (2)
C8	0.053 (3)	0.042 (3)	0.046 (3)	0.009 (2)	0.012 (2)	0.007 (2)
C9	0.042 (2)	0.038 (2)	0.039 (2)	0.0065 (19)	0.0125 (19)	0.0053 (19)
C10	0.062 (3)	0.049 (3)	0.052 (3)	0.001 (2)	0.028 (3)	0.012 (3)
C11	0.060 (3)	0.040 (3)	0.068 (4)	−0.002 (2)	0.012 (3)	0.004 (3)
C12	0.075 (4)	0.053 (3)	0.057 (4)	−0.011 (3)	−0.003 (3)	−0.001 (3)
C13	0.079 (4)	0.063 (4)	0.041 (3)	−0.010 (3)	0.003 (3)	0.009 (3)
C14	0.043 (3)	0.042 (3)	0.038 (3)	0.004 (2)	0.004 (2)	0.004 (2)

### Bromidobis(2,3-dihydro-1H-1,3-benzodiazole-2-thione)copper(I) (1) Geometric parameters (Å, º)

**Table:**

Br1—Cu1	2.4346 (8)	N4—C14	1.383 (6)
Cu1—S2	2.2189 (15)	N4—H4A	0.84 (4)
Cu1—S1	2.2464 (13)	C4—C5	1.386 (7)
S1—C1	1.688 (5)	C4—H4	0.95 (5)
N1—C1	1.356 (6)	C5—C6	1.364 (7)
N1—C2	1.384 (6)	C5—H5	0.92 (5)
N1—H1A	0.70 (4)	C6—C7	1.386 (6)
C1—N2	1.342 (5)	C6—H6	0.88 (5)
S2—C8	1.691 (5)	C9—C10	1.380 (6)
N2—C7	1.378 (5)	C9—C14	1.386 (6)
N2—H2A	0.82 (4)	C10—C11	1.365 (7)
C2—C3	1.371 (6)	C10—H10	0.82 (5)
C2—C7	1.387 (6)	C11—C12	1.378 (7)
N3—C8	1.350 (6)	C11—H11	0.97 (5)
N3—C9	1.381 (6)	C12—C13	1.368 (7)
N3—H3A	0.76 (4)	C12—H12	0.90 (5)
C3—C4	1.374 (7)	C13—C14	1.367 (7)
C3—H3	0.90 (4)	C13—H13	0.86 (4)
N4—C8	1.334 (6)		
			
S2—Cu1—S1	119.59 (5)	C6—C5—H5	121 (3)
S2—Cu1—Br1	121.07 (4)	C4—C5—H5	117 (3)
S1—Cu1—Br1	118.74 (4)	C5—C6—C7	116.8 (5)
C1—S1—Cu1	108.47 (15)	C5—C6—H6	124 (3)
C1—N1—C2	111.5 (4)	C7—C6—H6	119 (3)
C1—N1—H1A	120 (4)	N2—C7—C6	131.9 (4)
C2—N1—H1A	127 (4)	N2—C7—C2	107.1 (4)
N2—C1—N1	105.6 (4)	C6—C7—C2	121.0 (4)
N2—C1—S1	127.9 (3)	N4—C8—N3	105.8 (4)
N1—C1—S1	126.5 (3)	N4—C8—S2	128.4 (3)
C8—S2—Cu1	108.64 (16)	N3—C8—S2	125.8 (4)
C1—N2—C7	110.9 (4)	C10—C9—N3	133.5 (4)
C1—N2—H2A	127 (3)	C10—C9—C14	121.0 (4)
C7—N2—H2A	122 (3)	N3—C9—C14	105.5 (4)
C3—C2—N1	133.2 (4)	C11—C10—C9	117.6 (5)
C3—C2—C7	121.8 (4)	C11—C10—H10	124 (3)
N1—C2—C7	105.0 (4)	C9—C10—H10	118 (3)
C8—N3—C9	111.3 (4)	C10—C11—C12	121.1 (5)
C8—N3—H3A	129 (3)	C10—C11—H11	119 (3)
C9—N3—H3A	119 (3)	C12—C11—H11	120 (3)
C2—C3—C4	117.0 (5)	C13—C12—C11	121.6 (6)
C2—C3—H3	118 (3)	C13—C12—H12	121 (3)
C4—C3—H3	125 (3)	C11—C12—H12	117 (3)
C8—N4—C14	111.2 (4)	C14—C13—C12	117.7 (5)
C8—N4—H4A	120 (3)	C14—C13—H13	120 (3)
C14—N4—H4A	129 (3)	C12—C13—H13	122 (3)
C3—C4—C5	121.3 (5)	C13—C14—N4	132.8 (4)
C3—C4—H4	117 (3)	C13—C14—C9	121.0 (4)
C5—C4—H4	122 (3)	N4—C14—C9	106.2 (4)
C6—C5—C4	122.0 (5)		
			
C2—N1—C1—N2	0.9 (5)	C14—N4—C8—N3	1.7 (5)
C2—N1—C1—S1	179.3 (3)	C14—N4—C8—S2	−176.8 (3)
Cu1—S1—C1—N2	−11.7 (4)	C9—N3—C8—N4	−1.8 (5)
Cu1—S1—C1—N1	170.2 (3)	C9—N3—C8—S2	176.7 (3)
N1—C1—N2—C7	−0.8 (5)	Cu1—S2—C8—N4	−4.7 (5)
S1—C1—N2—C7	−179.2 (3)	Cu1—S2—C8—N3	177.1 (4)
C1—N1—C2—C3	178.4 (5)	C8—N3—C9—C10	−178.4 (5)
C1—N1—C2—C7	−0.7 (5)	C8—N3—C9—C14	1.2 (5)
N1—C2—C3—C4	−179.6 (5)	N3—C9—C10—C11	178.5 (5)
C7—C2—C3—C4	−0.7 (7)	C14—C9—C10—C11	−1.1 (7)
C2—C3—C4—C5	0.7 (8)	C9—C10—C11—C12	0.6 (8)
C3—C4—C5—C6	−0.3 (8)	C10—C11—C12—C13	0.0 (9)
C4—C5—C6—C7	−0.2 (8)	C11—C12—C13—C14	−0.2 (9)
C1—N2—C7—C6	−178.7 (5)	C12—C13—C14—N4	−178.5 (5)
C1—N2—C7—C2	0.4 (5)	C12—C13—C14—C9	−0.3 (7)
C5—C6—C7—N2	179.3 (5)	C8—N4—C14—C13	177.4 (5)
C5—C6—C7—C2	0.3 (7)	C8—N4—C14—C9	−1.0 (5)
C3—C2—C7—N2	−179.0 (4)	C10—C9—C14—C13	0.9 (7)
N1—C2—C7—N2	0.2 (4)	N3—C9—C14—C13	−178.7 (4)
C3—C2—C7—C6	0.2 (7)	C10—C9—C14—N4	179.5 (4)
N1—C2—C7—C6	179.4 (4)	N3—C9—C14—N4	−0.2 (5)

### Bromidobis(2,3-dihydro-1H-1,3-benzodiazole-2-thione)copper(I) (1) Hydrogen-bond geometry (Å, º)

**Table:**

*D*—H···*A*	*D*—H	H···*A*	*D*···*A*	*D*—H···*A*
N1—H1*A*···S1^i^	0.70 (4)	2.69 (4)	3.384 (4)	170 (5)
N2—H2*A*···Br1	0.82 (4)	2.65 (4)	3.361 (4)	146 (4)
N4—H4*A*···Br1	0.84 (4)	2.54 (4)	3.364 (4)	166 (4)

Symmetry code: (i) −*x*+1, −*y*+1, −*z*+1.

### Bis(2,3-dihydro-1H-1,3-benzodiazole-2-thione)iodidocopper(I) acetone solvate (2) Crystal data

**Table:**

[CuI(C_7_H_6_N_2_S)_2_]·C_3_H_6_O	*F*(000) = 1080
*M**_r_* = 548.91	*D*_x_ = 1.787 Mg m^−^^3^
Monoclinic, *P*2_1_/*c*	Mo *K*α radiation, λ = 0.71073 Å
*a* = 4.5154 (3) Å	Cell parameters from 5872 reflections
*b* = 22.2157 (15) Å	θ = 2.2–26.6°
*c* = 20.4062 (14) Å	µ = 2.80 mm^−^^1^
β = 94.818 (1)°	*T* = 293 K
*V* = 2039.8 (2) Å^3^	Needle, colourless
*Z* = 4	0.38 × 0.14 × 0.08 mm

### Bis(2,3-dihydro-1H-1,3-benzodiazole-2-thione)iodidocopper(I) acetone solvate (2) Data collection

**Table:**

Bruker CCD area detector diffractometer	3597 independent reflections
Radiation source: fine-focus sealed tube	3200 reflections with *I* > 2σ(*I*)
Graphite monochromator	*R*_int_ = 0.021
Frames, each covering 0.3 ° in ω scans	θ_max_ = 25.0°, θ_min_ = 1.4°
Absorption correction: multi-scan (SADABS; Bruker, 2003)	*h* = −5→5
*T*_min_ = 0.749, *T*_max_ = 1.000	*k* = −26→26
14555 measured reflections	*l* = −24→24

### Bis(2,3-dihydro-1H-1,3-benzodiazole-2-thione)iodidocopper(I) acetone solvate (2) Refinement

**Table:**

Refinement on *F*^2^	Primary atom site location: structure-invariant direct methods
Least-squares matrix: full	Secondary atom site location: difference Fourier map
*R*[*F*^2^ > 2σ(*F*^2^)] = 0.039	Hydrogen site location: mixed
*wR*(*F*^2^) = 0.089	H atoms treated by a mixture of independent and constrained refinement
*S* = 1.08	*w* = 1/[σ^2^(*F*_o_^2^) + (0.0315*P*)^2^ + 4.5225*P*] where *P* = (*F*_o_^2^ + 2*F*_c_^2^)/3
3597 reflections	(Δ/σ)_max_ = 0.002
247 parameters	Δρ_max_ = 1.14 e Å^−^^3^
4 restraints	Δρ_min_ = −0.90 e Å^−^^3^

### Bis(2,3-dihydro-1H-1,3-benzodiazole-2-thione)iodidocopper(I) acetone solvate (2) Special details

**Table:**

Geometry. All esds (except the esd in the dihedral angle between two l.s. planes) are estimated using the full covariance matrix. The cell esds are taken into account individually in the estimation of esds in distances, angles and torsion angles; correlations between esds in cell parameters are only used when they are defined by crystal symmetry. An approximate (isotropic) treatment of cell esds is used for estimating esds involving l.s. planes.

### Bis(2,3-dihydro-1H-1,3-benzodiazole-2-thione)iodidocopper(I) acetone solvate (2) Fractional atomic coordinates and isotropic or equivalent isotropic displacement parameters (Å^2^)

**Table:**

	*x*	*y*	*z*	*U*_iso_*/*U*_eq_	
Cu1	0.2323 (2)	0.42788 (3)	0.63559 (3)	0.0789 (2)	
I1	0.34973 (7)	0.31632 (2)	0.65076 (2)	0.06112 (13)	
S1	−0.1755 (4)	0.45699 (6)	0.57174 (7)	0.0838 (5)	
S2	0.3444 (3)	0.50247 (5)	0.70750 (6)	0.0518 (3)	
N1	−0.1238 (9)	0.35326 (17)	0.50250 (18)	0.0544 (9)	
N2	−0.4365 (10)	0.41666 (16)	0.45551 (19)	0.0555 (10)	
N3	0.7524 (8)	0.42258 (15)	0.76321 (17)	0.0454 (8)	
N4	0.6946 (8)	0.50239 (16)	0.82129 (18)	0.0483 (8)	
C1	−0.2443 (12)	0.4079 (2)	0.5087 (2)	0.0548 (11)	
C2	−0.2433 (10)	0.32568 (19)	0.4450 (2)	0.0476 (10)	
C3	−0.1898 (11)	0.2710 (2)	0.4161 (2)	0.0557 (11)	
H3	−0.054352	0.243569	0.435797	0.067*	
C4	−0.3470 (11)	0.2590 (2)	0.3568 (2)	0.0592 (12)	
H4	−0.316081	0.222687	0.335867	0.071*	
C5	−0.5483 (12)	0.2993 (2)	0.3277 (2)	0.0610 (13)	
H5	−0.651343	0.289205	0.287859	0.073*	
C6	−0.6020 (12)	0.3539 (2)	0.3555 (2)	0.0579 (12)	
H6	−0.737871	0.381133	0.335589	0.070*	
C7	−0.4427 (10)	0.36649 (18)	0.4150 (2)	0.0479 (10)	
C8	0.6017 (9)	0.47473 (18)	0.7644 (2)	0.0442 (10)	
C9	0.9438 (9)	0.41641 (18)	0.8198 (2)	0.0438 (9)	
C10	1.1416 (10)	0.3719 (2)	0.8413 (2)	0.0541 (11)	
H10	1.170348	0.337767	0.816134	0.065*	
C11	1.2951 (11)	0.3804 (2)	0.9021 (3)	0.0619 (13)	
H11	1.428668	0.351140	0.918471	0.074*	
C12	1.2554 (11)	0.4315 (2)	0.9394 (2)	0.0620 (13)	
H12	1.362526	0.435638	0.980133	0.074*	
C13	1.0610 (10)	0.4761 (2)	0.9175 (2)	0.0550 (11)	
H13	1.035593	0.510598	0.942271	0.066*	
C14	0.9046 (9)	0.46766 (19)	0.8569 (2)	0.0458 (10)	
C15	0.1969 (15)	0.6773 (3)	0.9353 (3)	0.0795 (17)	
H15A	0.041024	0.704543	0.919993	0.119*	
H15B	0.126452	0.650874	0.967768	0.119*	
H15C	0.363937	0.699853	0.954390	0.119*	
C16	0.2888 (11)	0.6414 (2)	0.8792 (2)	0.0553 (11)	
C17	0.1359 (14)	0.6534 (3)	0.8138 (3)	0.0797 (16)	
H17A	−0.010436	0.684287	0.817434	0.120*	
H17B	0.278159	0.666406	0.784409	0.120*	
H17C	0.040343	0.617277	0.797060	0.120*	
O1	0.4835 (9)	0.60411 (17)	0.88765 (19)	0.0792 (11)	
H1A	−0.007 (11)	0.339 (3)	0.534 (2)	0.095*	
H2A	−0.543 (12)	0.4484 (18)	0.449 (3)	0.095*	
H3A	0.733 (14)	0.398 (2)	0.731 (2)	0.095*	
H4A	0.617 (13)	0.5354 (16)	0.832 (3)	0.095*	

### Bis(2,3-dihydro-1H-1,3-benzodiazole-2-thione)iodidocopper(I) acetone solvate (2) Atomic displacement parameters (Å^2^)

**Table:**

	*U*^11^	*U*^22^	*U*^33^	*U*^12^	*U*^13^	*U*^23^
Cu1	0.1375 (7)	0.0464 (4)	0.0480 (3)	0.0166 (4)	−0.0207 (4)	−0.0050 (3)
I1	0.0590 (2)	0.03985 (18)	0.0816 (3)	0.00121 (13)	−0.01114 (16)	−0.00181 (14)
S1	0.1445 (14)	0.0479 (7)	0.0528 (7)	0.0360 (8)	−0.0277 (8)	−0.0143 (6)
S2	0.0627 (7)	0.0405 (6)	0.0517 (6)	0.0037 (5)	0.0028 (5)	−0.0012 (5)
N1	0.077 (3)	0.040 (2)	0.045 (2)	0.0109 (19)	−0.0036 (19)	−0.0014 (16)
N2	0.078 (3)	0.039 (2)	0.049 (2)	0.0103 (19)	−0.0031 (19)	−0.0006 (17)
N3	0.049 (2)	0.0382 (19)	0.050 (2)	0.0005 (16)	0.0054 (16)	−0.0060 (15)
N4	0.057 (2)	0.039 (2)	0.049 (2)	0.0016 (17)	0.0093 (17)	−0.0059 (17)
C1	0.083 (3)	0.039 (2)	0.042 (2)	0.009 (2)	0.001 (2)	−0.0012 (19)
C2	0.061 (3)	0.040 (2)	0.043 (2)	−0.003 (2)	0.012 (2)	0.0001 (18)
C3	0.066 (3)	0.041 (2)	0.062 (3)	0.004 (2)	0.014 (2)	−0.005 (2)
C4	0.077 (3)	0.044 (3)	0.059 (3)	−0.011 (2)	0.018 (3)	−0.014 (2)
C5	0.078 (3)	0.055 (3)	0.050 (3)	−0.018 (3)	0.006 (2)	−0.007 (2)
C6	0.072 (3)	0.049 (3)	0.051 (3)	−0.008 (2)	−0.002 (2)	0.004 (2)
C7	0.064 (3)	0.037 (2)	0.043 (2)	−0.004 (2)	0.010 (2)	0.0021 (18)
C8	0.045 (2)	0.040 (2)	0.049 (2)	−0.0065 (18)	0.0120 (19)	−0.0023 (18)
C9	0.043 (2)	0.042 (2)	0.047 (2)	−0.0049 (18)	0.0097 (18)	−0.0012 (18)
C10	0.055 (3)	0.049 (3)	0.060 (3)	0.002 (2)	0.010 (2)	−0.004 (2)
C11	0.058 (3)	0.063 (3)	0.065 (3)	0.009 (2)	0.005 (2)	0.007 (2)
C12	0.060 (3)	0.077 (3)	0.049 (3)	−0.002 (3)	0.003 (2)	−0.001 (2)
C13	0.060 (3)	0.057 (3)	0.048 (3)	−0.003 (2)	0.009 (2)	−0.008 (2)
C14	0.045 (2)	0.043 (2)	0.051 (2)	−0.0016 (19)	0.0104 (19)	−0.0014 (19)
C15	0.100 (4)	0.072 (4)	0.067 (3)	0.025 (3)	0.007 (3)	−0.006 (3)
C16	0.063 (3)	0.046 (3)	0.057 (3)	0.001 (2)	0.007 (2)	−0.004 (2)
C17	0.088 (4)	0.088 (4)	0.064 (3)	0.006 (3)	0.006 (3)	0.001 (3)
O1	0.094 (3)	0.065 (2)	0.077 (2)	0.029 (2)	−0.002 (2)	−0.0166 (19)

### Bis(2,3-dihydro-1H-1,3-benzodiazole-2-thione)iodidocopper(I) acetone solvate (2) Geometric parameters (Å, º)

**Table:**

Cu1—S2	2.2430 (13)	C5—C6	1.369 (7)
Cu1—S1	2.2594 (17)	C5—H5	0.9300
Cu1—I1	2.5479 (7)	C6—C7	1.386 (6)
S1—C1	1.696 (4)	C6—H6	0.9300
S2—C8	1.689 (4)	C9—C10	1.380 (6)
N1—C1	1.341 (6)	C9—C14	1.387 (6)
N1—C2	1.391 (6)	C10—C11	1.383 (7)
N1—H1A	0.86 (2)	C10—H10	0.9300
N2—C1	1.345 (6)	C11—C12	1.386 (7)
N2—C7	1.387 (5)	C11—H11	0.9300
N2—H2A	0.86 (2)	C12—C13	1.374 (7)
N3—C8	1.345 (5)	C12—H12	0.9300
N3—C9	1.390 (5)	C13—C14	1.384 (6)
N3—H3A	0.85 (2)	C13—H13	0.9300
N4—C8	1.348 (5)	C15—C16	1.484 (7)
N4—C14	1.381 (6)	C15—H15A	0.9600
N4—H4A	0.85 (2)	C15—H15B	0.9600
C2—C3	1.381 (6)	C15—H15C	0.9600
C2—C7	1.384 (6)	C16—O1	1.209 (6)
C3—C4	1.376 (7)	C16—C17	1.474 (7)
C3—H3	0.9300	C17—H17A	0.9600
C4—C5	1.375 (7)	C17—H17B	0.9600
C4—H4	0.9300	C17—H17C	0.9600
			
S2—Cu1—S1	107.10 (5)	N2—C7—C6	131.8 (4)
S2—Cu1—I1	127.30 (4)	N3—C8—N4	106.7 (4)
S1—Cu1—I1	119.98 (5)	N3—C8—S2	128.4 (3)
C1—S1—Cu1	110.01 (18)	N4—C8—S2	124.9 (3)
C8—S2—Cu1	106.54 (15)	C10—C9—C14	121.6 (4)
C1—N1—C2	110.3 (4)	C10—C9—N3	132.5 (4)
C1—N1—H1A	120 (4)	C14—C9—N3	105.9 (4)
C2—N1—H1A	129 (4)	C9—C10—C11	116.6 (4)
C1—N2—C7	110.1 (4)	C9—C10—H10	121.7
C1—N2—H2A	124 (4)	C11—C10—H10	121.7
C7—N2—H2A	126 (4)	C10—C11—C12	121.8 (5)
C8—N3—C9	110.5 (3)	C10—C11—H11	119.1
C8—N3—H3A	123 (4)	C12—C11—H11	119.1
C9—N3—H3A	126 (4)	C13—C12—C11	121.3 (5)
C8—N4—C14	110.4 (4)	C13—C12—H12	119.3
C8—N4—H4A	121 (4)	C11—C12—H12	119.3
C14—N4—H4A	128 (4)	C12—C13—C14	117.2 (4)
N1—C1—N2	107.1 (4)	C12—C13—H13	121.4
N1—C1—S1	127.1 (4)	C14—C13—H13	121.4
N2—C1—S1	125.7 (3)	N4—C14—C13	132.1 (4)
C3—C2—C7	121.2 (4)	N4—C14—C9	106.6 (4)
C3—C2—N1	132.7 (4)	C13—C14—C9	121.4 (4)
C7—C2—N1	106.1 (4)	C16—C15—H15A	109.5
C4—C3—C2	116.7 (5)	C16—C15—H15B	109.5
C4—C3—H3	121.7	H15A—C15—H15B	109.5
C2—C3—H3	121.7	C16—C15—H15C	109.5
C5—C4—C3	121.9 (4)	H15A—C15—H15C	109.5
C5—C4—H4	119.1	H15B—C15—H15C	109.5
C3—C4—H4	119.1	O1—C16—C17	122.2 (5)
C6—C5—C4	122.1 (5)	O1—C16—C15	120.4 (5)
C6—C5—H5	119.0	C17—C16—C15	117.4 (5)
C4—C5—H5	119.0	C16—C17—H17A	109.5
C5—C6—C7	116.4 (5)	C16—C17—H17B	109.5
C5—C6—H6	121.8	H17A—C17—H17B	109.5
C7—C6—H6	121.8	C16—C17—H17C	109.5
C2—C7—N2	106.4 (4)	H17A—C17—H17C	109.5
C2—C7—C6	121.7 (4)	H17B—C17—H17C	109.5
			
C2—N1—C1—N2	−1.0 (6)	C9—N3—C8—N4	0.1 (5)
C2—N1—C1—S1	178.0 (4)	C9—N3—C8—S2	−179.9 (3)
C7—N2—C1—N1	0.9 (6)	C14—N4—C8—N3	−0.3 (5)
C7—N2—C1—S1	−178.2 (4)	C14—N4—C8—S2	179.6 (3)
Cu1—S1—C1—N1	13.2 (5)	Cu1—S2—C8—N3	10.3 (4)
Cu1—S1—C1—N2	−168.0 (4)	Cu1—S2—C8—N4	−169.7 (3)
C1—N1—C2—C3	178.4 (5)	C8—N3—C9—C10	−180.0 (5)
C1—N1—C2—C7	0.8 (5)	C8—N3—C9—C14	0.2 (5)
C7—C2—C3—C4	−0.8 (7)	C14—C9—C10—C11	1.0 (7)
N1—C2—C3—C4	−178.1 (5)	N3—C9—C10—C11	−178.8 (4)
C2—C3—C4—C5	−0.2 (7)	C9—C10—C11—C12	−0.7 (7)
C3—C4—C5—C6	0.8 (8)	C10—C11—C12—C13	−0.1 (8)
C4—C5—C6—C7	−0.2 (7)	C11—C12—C13—C14	0.6 (7)
C3—C2—C7—N2	−178.2 (4)	C8—N4—C14—C13	−179.3 (5)
N1—C2—C7—N2	−0.3 (5)	C8—N4—C14—C9	0.4 (5)
C3—C2—C7—C6	1.4 (7)	C12—C13—C14—N4	179.3 (5)
N1—C2—C7—C6	179.3 (4)	C12—C13—C14—C9	−0.3 (7)
C1—N2—C7—C2	−0.4 (5)	C10—C9—C14—N4	179.8 (4)
C1—N2—C7—C6	−179.9 (5)	N3—C9—C14—N4	−0.4 (4)
C5—C6—C7—C2	−0.8 (7)	C10—C9—C14—C13	−0.5 (7)
C5—C6—C7—N2	178.6 (5)	N3—C9—C14—C13	179.4 (4)

### Bis(2,3-dihydro-1H-1,3-benzodiazole-2-thione)iodidocopper(I) acetone solvate (2) Hydrogen-bond geometry (Å, º)

**Table:**

*D*—H···*A*	*D*—H	H···*A*	*D*···*A*	*D*—H···*A*
N1—H1*A*···I1	0.86 (2)	2.81 (2)	3.649 (4)	167 (6)
N2—H2*A*···S1^i^	0.86 (2)	2.47 (2)	3.331 (4)	176 (6)
N3—H3*A*···I1	0.85 (2)	2.91 (4)	3.666 (3)	148 (5)
N4—H4*A*···O1	0.85 (2)	2.02 (3)	2.840 (5)	161 (6)
C15—H15*A*···I1^ii^	0.96	3.31	4.240 (6)	165

Symmetry codes: (i) −*x*−1, −*y*+1, −*z*+1; (ii) −*x*, *y*+1/2, −*z*+3/2.
